# Comparative Study of Tributyltin Adsorption onto Mesoporous Silica Functionalized with Calix[4]arene, *p*-*tert*-Butylcalix[4]arene and *p*-Sulfonatocalix[4]arene

**DOI:** 10.3390/molecules19044524

**Published:** 2014-04-10

**Authors:** Sana Alahmadi, Sharifah Mohamad, Mohd Jamil Maah

**Affiliations:** 1 Department of Chemistry, Faculty of Science, University Malaya, Kuala Lumpur 50603, Malaysia; E-Mails: sharifahm@um.edu.my (S.M.); mjamil@um.edu.my (M.J.M.); 2 Department of Chemistry, Faculty of Science, Taibah University, Almadina Almonwara 30001, Saudi Arabia

**Keywords:** tributyltin, functionalized MCM-41, adsorption isotherm, thermodynamics

## Abstract

The adsorption of tributyltin (TBT), onto three mesoporous silica adsorbents functionalized with calix[4]arene, *p-tert*-butylcalix[4]arene and *p*-sulfonatocalix[4]arene (MCM-TDI-C4, MCM-TDI-PC4 and MCM-TDI-C4S, respectively) has been compared. Batch adsorption experiments were carried out and the effect of contact time, initial TBT concentration, pH and temperature were studied. The Koble–Corrigan isotherm was the most suitable for data fitting. Based on a Langmuir isotherm model, the maximum adsorption capacities were 12.1212, 16.4204 and 7.5757 mg/g for MCM-TDI-C4, MCM-TDI-PC4 and MCM-TDI-C4S, respectively. The larger uptake and stronger affinity of MCM-TDI-PC4 than MCM-TDI-C4 and MCM-TDI-C4S probably results from van der Waals interactions and the pore size distribution of MCM-TDI-PC4. Gibbs free energies for the three adsorption processes of TBT presented a negative value, reflecting that TBT/surface interactions are thermodynamic favorable and spontaneous. The interaction processes were accompanied by an increase of entropy value for MCM-TDI-C4 and MCM-TDI-C4S (43.7192 and 120.7609 J/mol K, respectively) and a decrease for MCM-TDI-PC4 (−37.4704 J/mol K). It is obviously observed that MCM-TDI-PC4 spontaneously adsorbs TBT driven mainly by enthalpy change, while MCM-TDI-C4 and MCM-TDI-C4S do so driven mainly by entropy changes.

## 1. Introduction

Organotin compounds (OTCs) are the most commonly used organometallic compounds in agricultural, industrial and biomedical applications like PVC stabilizers, industrial catalysts, wood preservatives, fungicides and pesticides [1,2]. Nevertheless, their notoriety probably stems from the use of tributyltin (TBT) as the active biocide in antifouling paints. TBT is one of the most toxic compounds that is deliberately released into the environment [3] and is referred to as a potent endocrine disruptor exhibiting immunotoxic and genotoxic capabilities to a wide range of organisms from bacteria to human beings [4]. 

The adverse effects of TBT on aquatic ecosystems, in the worst cases, are accompanied by the extinction of local mollusc populations [5], and has eventually led to its usage restriction. Following several unsuccessful legislative measures [6], TBT was consequently banned in Europe in 2003 (Directive 2002/62/EC) and in all countries in 2008, with the enforcement of the “International Convention on the Control of Harmful Antifouling Systems”. However, even with this ban, OTC levels in sediments are still quite high due to the TBT’s affinity for particulate matter and its slow process of degradation under anaerobic conditions [1]. 

Various processes have been proposed to remove TBT through conventional treatment technologies such as biodegradation [7,8,9,10] and adsorption process. Adsorption using sorbents is among the widely employed methods to remove pollutants. Systematic examinations of the adsorption of TBT on various absorbents like natural sediments [11,12,13], pure minerals [14,15], organic matters [16] and black carbons [13,17] have been carried out. 

The adsorption technique is considered to be among the most effective approaches to remove pollutants from effluents. The process is more advantageous than other methods because of the flexibility of the system, low energy consumption and low operating costs. Recent publications concerning the adsorption of toxic compounds have revealed an increasing interest in the synthesis of adsorbents capable of completely eliminating organic pollutants. In this regard, supramolecular chemistry has offered more a effective solution to determine molecular structures that can serve as building blocks for the production of sophisticated molecules by anchoring functional groups in a manner that they offer an appropriate binding site. This was brought about by the creation of macrocyclic molecules, including synthetic crown ethers, cryptands, spherands [18], natural cyclodextrins [19] and calixarenes [20,21,22,23].

Recently, the promising synthetic materials calixerenes have received much interest due to their recognition of harmful inorganic and organic pollutants. Data on the sorption separation of tributyltin from aqueous solution onto organic-inorganic hybrid materials are limited and no work describing the use of mesoporous silica functionalized with calixarene for the adsorption and separation of tributyltin has been published. Furthermore, no work about the isotherm modeling, kinetics and thermodynamics of adsorption of tributyltin using supramolecular compounds as adsorbent has been reported.

**Figure 1:**
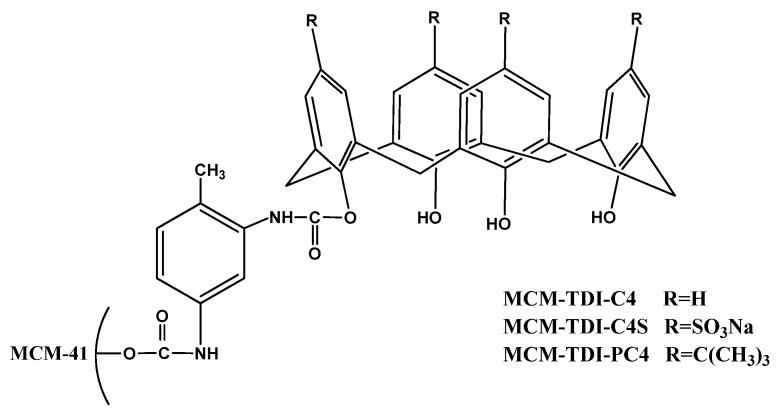
Chemical structures of the synthetic adsorbents.

The objective of this work was to investigate and compare the adsorption efficiency of some prepared sorbents, MCM-TDI-C4, MCM-TDI-PC4 and MCM-TDI-C4S (Figure 1), and specifically to investigate the adsorption behavior of TBT on these prepared sorbents. The impact of the physico-chemical properties of the three adsorbents, such as pore size distribution and surface functional groups on TBT uptake are discussed.

## 2. Results and Discussion

### 2.1. Characterization of Functionalized Mesoporous Silica with Calix[4]arene Derivatives

#### 2.1.1. Fourier Transform Infrared Spectroscopy (FTIR)

**Figure 2:**
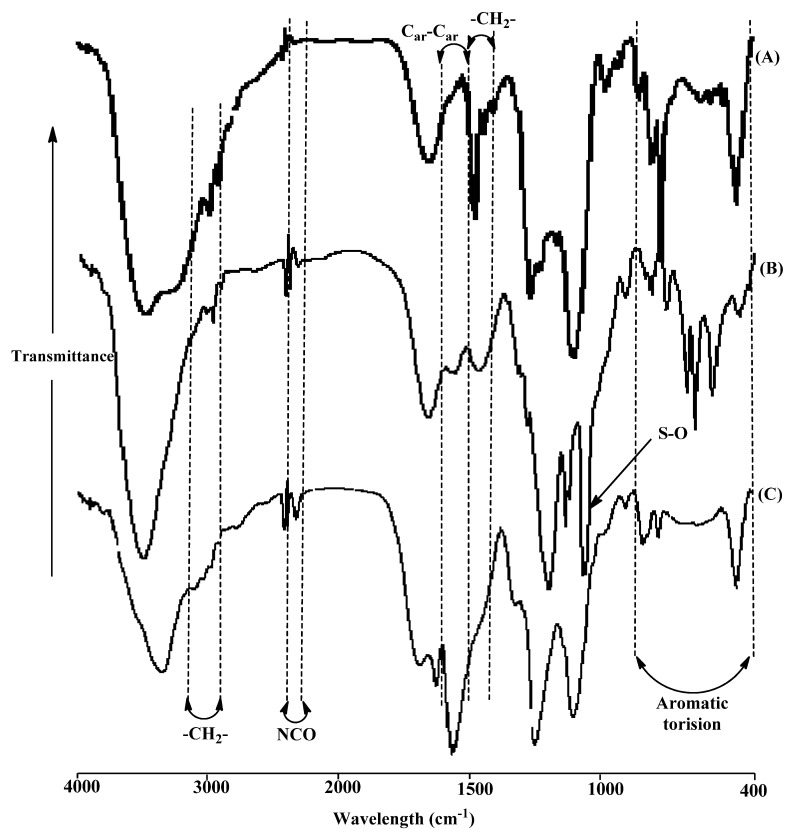
FTIR spectra of MCM-TDI-C4 (**A**), MCM-TDI-C4S (**B**) and MCM-TDI-PC4 (**C**).

Three mesoporous silica modified with calix[4]arene derivatives have been prepared via functionalization of modified mesoporous silica with toluene-2,4-diisocyanate (TDI) as linker and C4, C4S and PC4 as organic modifier. Toluene-2,4-diisocyanate was utilized to establish a bridge between the surface of mesoporous silica and calix[4]arene derivatives. These functionalization reactions were examined by FTIR spectroscopy to monitor the appearance and disappearance of some peaks. In detail, MCM-TDI-C4 (Figure 2A) presents a strong band at 3,423 cm^−1^ and its shoulder near 3,198 cm^−1^, which correspond to the -OH group of the mesoporous silica surface and the aromatic OH, respectively. There were two bands at 1,420 and 1,379 cm^−1^, and both bands seem to belong to COH bending vibration. The medium-intensity peak and the weak intensity peaks at 1,449 cm^−1^, 2,941 and 2,862 cm^−1^, respectively, corresponds to methylene bridges –CH_2_–. The band at 1,078–1,229 cm^−1^ which was referred to Si–O–Si, was broadened with C_ar_-O stretching at 1,241 cm^−1^. The bands at 807 and 755 cm^−1^ were related to aromatic torsion vibrations [24]. The band at 487 cm^−1^ may be assigned to the macrocycle torsion [24].

The spectrum of MCM-TDI-C4S (Figure 2B) shows three main bands at 3,449, 1,446 and 1,051 cm^−1^ assigned to the N–H and -OH groups of both hydroxides for the mesoporous silica and the C4S molecule, the weak absorption peak of methylene bridges –CH_2_– and the strong absorption peak of S–O which broadened the peak of Si–O–Si, respectively.

From Figure 2C, it can be seen that the spectrum of MCM-TDI-PC4 presents a strong band at 1,542 cm^−1^ and its shoulder near 1,424 cm^−1^, which correspond to the phenyl *v*C_ar_–C_ar_ and methylene bridges –CH_2_–, respectively. The results were closely in agreement with the published data [25,26] and indicated that *p-tert*-butylcalix[4]arene was successfully bonded on the surface.

The efficiency of the grafting process was demonstrated by a significant decrease in the isocyanate group band at around 2,270 cm^−1^, with an associated increase of characteristic new bands of the immobilized calix[4]arene derivatives. Meanwhile, the absorption at 2,275 cm^−1^ in the spectra of MCM-TDI-C4 and MCM-TDI-C4S disappeared. This indicates that the calix[4]arene derivatives were successfully bonded on the surface. However, in the case of MCM-TDI-PC4, the weak absorption peak of the isocyanate group still appears and this may be due to the steric hindrance.

#### 2.1.2. Nitrogen Adsorption-Desorption Measurements

**Figure 3:**
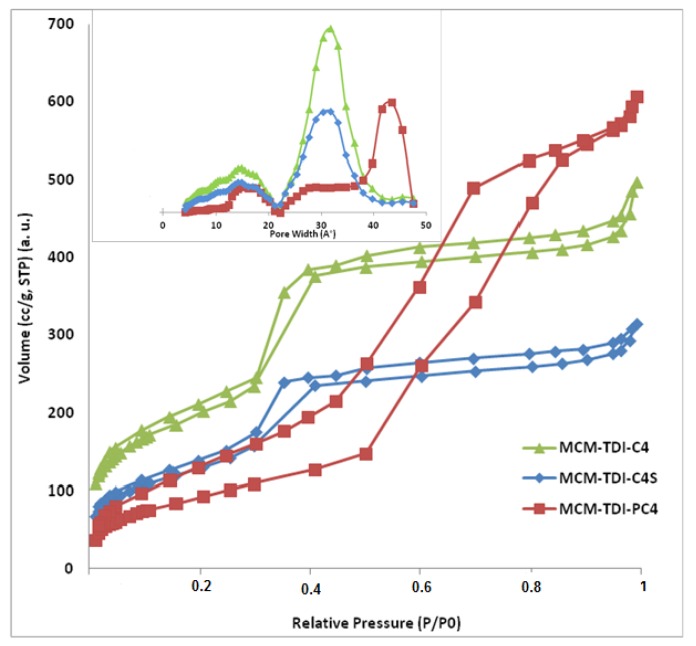
Nitrogen adsorption-desorption isotherms and pore size distribution of MCM**-**TDI**-**C4, MCM**-**TDI**-**C4S, and MCM**-**TDI**-**PC4.

In order to investigate the channel structure of the prepared materials, the characterization of the nitrogen adsorption-desorption was carried out. The corresponding isotherms are presented in Figure 3. They all exhibit the typical Type IV isotherms according to the IUPAC classification [27], which correspond to the characteristics of mesoporous materials with highly uniform size distributions. 

The BET isotherms of the modified samples MCM-TDI-C4 and MCM-TDI-C4S in Figure 3 show small hysteresis loops of type H1, which is a typical characteristic of mesoporous materials with well-defined cylindrical-like pore channels [28]. In case of MCM-TDI-PC4, the BET isotherm shows hysteresis loops of type H2, which describes materials that are frequently disordered with not well-defined pore size and shape indicating bottleneck constrictions [28]. This may be due to the bulky organic group located inside the pore channel [29,30,31]. The structure data of modified mesoporous materials MCM-TDI-C4, MCM-TDI-C4S and MCM-TDI-PC4 (surface area, total pore volume, and pore diameter) are summarized in Table 1.

**Table 1:** Structural parameters of synthetic adsorbents.

Sample	S_BET_ (m^2^/g)	V (cm^3^/g)	D (nm)
MCM-TDI-C4	733	0.67	3.6
MCM-TDI-C4S	452	0.43	3.8
MCM-TDI-PC4	339	0.32	3.9

### 2.2. Effect of Contact Time

**Figure 4:**
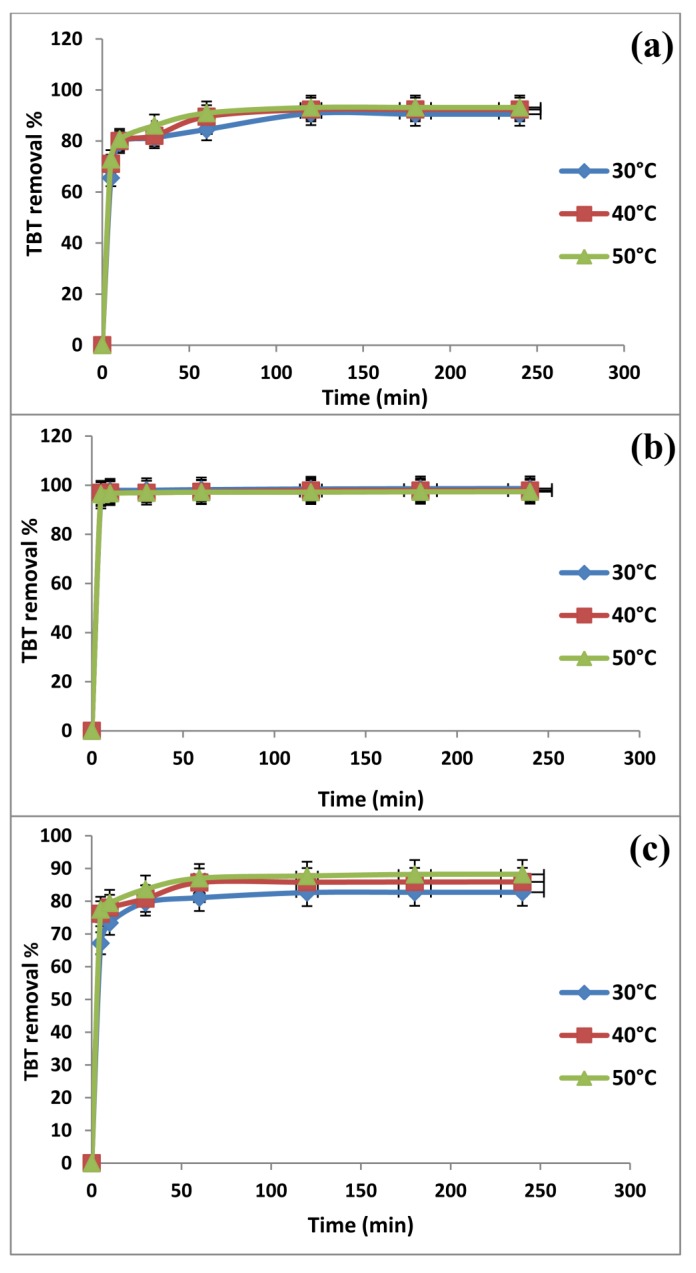
Effect of contact time on removal of TBT onto MCM-TDI-C4 (**a**), MCM-TDI-PC4 (**b**) and MCM-TDI-C4S (**c**).

Prior to conducting the batch uptake equilibrium experiments, the determination of contact time needed for adsorption equilibrium was required. The TBT uptakes by the synthesized mesoporous silica MCM-TDI-C4, MCM-TDI-PC4 and MCM-TDI-C4S are shown in Figure 4 as a function of contact time at 30, 40 and 50 °C. It is apparent from Figure 4 that in the first 10 min, the percentage of removal of TBT from aqueous solution increased rapidly and reached 81%, 98% and 80% for MCM-TDI-C4, MCM-TDI-PC4 and MCM-TDI-C4S, respectively. After that, the percentage of removal of TBT increased slowly until 120 min and then subsequently became constant. The results indicated that the rate of adsorption of TBT was faster during the initial time of adsorption and has less effect on the rate of adsorption in the latter half of the process. This was due to the nature of the adsorbent and the available adsorption sites, which affect the rate of adsorption of TBT. This difference in the rate of adsorption may be due to the fact that initially, all adsorbent sites were vacant so the adsorption was high. Later, due to the decreased in the number of adsorption sites on the adsorbents, as well as TBT concentration, the adsorption of TBT became slow. Furthermore, the remaining vacant surface sites were difficult to be occupied due to the repulsive forces, as well as the competition between TBT molecules on the adsorbent surface [32]. The equilibrium time required for the adsorption of TBT was 2 h for all adsorbents. Prior literature provided varying duration of equilibrium in batch experiments. For instance, Unger *et al*. [33] revealed that the equilibrium state for TBT was reached after a few minutes to a few hours, while Langston and Pope [34] reposted that 85.7% of the added TBT amount was adsorbed onto the solid phase in 2 h. 

The highest removal percentage of TBT was obtained with MCM-TDI-PC4. Although S_BET_ for MCM-TDI-PC4 was lower (339 m^2^/g) in comparison with other adsorbents, its adsorption capacity was higher. This can be attributed to the nature of the adsorbent, including the surface area, pore size distribution, hydrophobicity, which is defined as the concentration of carbon atoms in the matrix, and the density and type of functional groups present on the surface [35]. The first layer capacity was defined as the calixarene loading, while the second layer inherently represents all external adsorption sites, including those on the residual oxide surface, as well as any non-cavity sites associated with calixarenes (for example, on the outer surface of the *tert*-butyl groups). The latter also includes interstitial sites between adjacent grafted calixarenes [36]. A comparison of the materials MCM-TDI-C4, MCM-TDI-PC4 and MCM-TDI-C4S, in which the only difference in calixarene structure is the R group, shows that the uptake increased as the size of the hydrophobic R group changes from H to *tert*-butyl. This suggests that the upper-rim calixarene functional groups contribute substantially to the degree of uptake, but the pore diameter could also play an important role. These results suggested that differences in the adsorption between materials were largely influence by the non-specific van der Waals interactions occurring between the *n*-butyl chain of the butyl group in TBT, as well as the alkyl R groups on the calixarene upper rim. This is consistent with the results found by Thompson *et al*. [36].

Furthermore, the higher TBT capacities of the MCM-TDI-PC4 compared to MCM-TDI-C4 and MCM-TDI-C4S can be explained by the fact that the MCM-TDI-PC4 contains a broader pore size distribution (Figure 3). The nitrogen sorption isotherms (Figure 3) were Type IV for all samples confirming their mesoporous nature. However slightly different hysteresis loops were noted for MCM-TDI-PC4 compared with MCM-TDI-C4 and MCM-TDI-C4S. The broader hysteresis loop for MCM-TDI-PC4 suggested that this material contained pores with different shapes and size [37]. Despite having a similar average pore size to MCM-TDI-C4 and MCM-TDI-C4S, MCM-TDI-PC4 differed in a sense that it has a broader pore size distribution.

### 2.3. Influence of pH on Removal Efficiency of TBT

**Figure 5:**
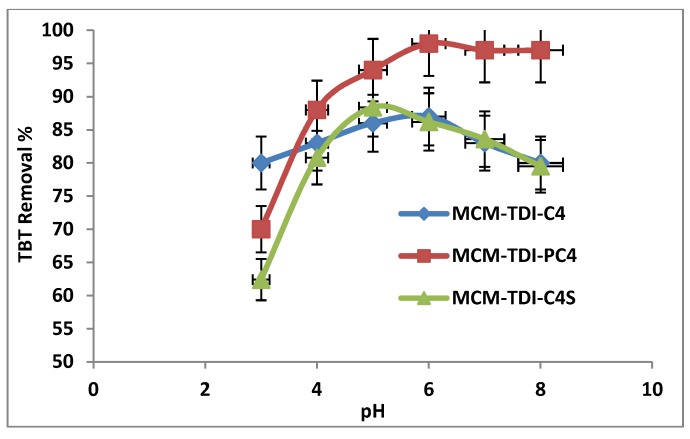
Effect of pH on removal of TBT.

System pH during adsorption has an important role on the surface characteristics of the adsorbent particles, and consequently, overall adsorption performance. Illustrated in Figure 5 is the effect of pH on TBT adsorption onto MCM-TDI-C4, MCM-TDI-PC4 and MCM-TDI-C4S. The adsorption behaviour can be explained through the TBT species and the functional group existing in the surface of the adsorbent. TBT is characterized as a weak acid having a pKa of 6.3 [38] comprising of cationic form (TBT^+^, (C_4_H_9_)_3_Sn) in equilibrium with a neutral form (TBTOH, (C_4_H_9_)_3_SnOH) as evident from Equation (1):

[TBT^+^ + H_2_O ⇄ TBTOH + H^+^] (1)

At pH < pKa, the adsorption process of TBT^+^ is governed by electrostatic attraction. However, it is postulated that the major driving force of adsorption at pH > pKa is the hydrophobic character of the TBT compound, which is less effective than the electrostatic interaction [39]. With a pH of over 6, the main species is TBTOH, while the predominant species of tributyltin at acidic pH is TBT^+^. However, under acidic conditions, adsorbent surface becomes a significantly protonated surface that goes against the uptake of TBT^+^ form due to electrostatic repulsion.

The TBT maximum adsorption took place at a medium pH for all adsorbents, and this is in analogous to the TBT adsorption by the beech charcoal and soot black carbon [17]. At higher pH (pH > pKa), the adsorption decreased due to the TBT species (TBTOH), which are not favorable for those three materials.

**Scheme 1:**
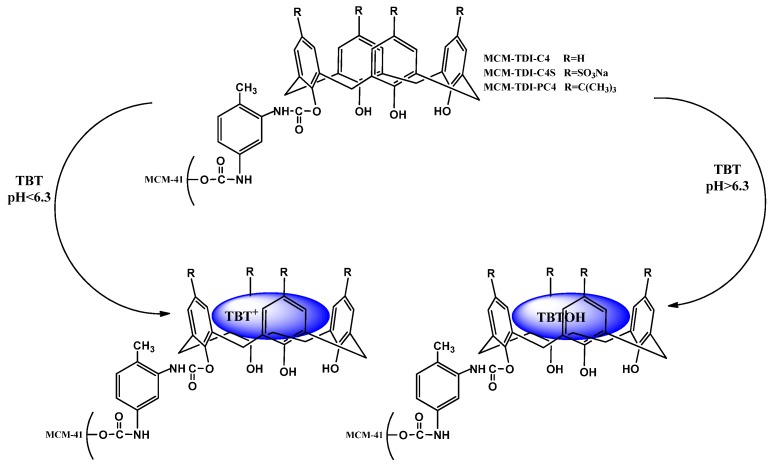
Proposed interaction of tributyltin with adsorbents.

It was observed that the anchoring of calixarenes onto solid surfaces played an important role in governing both the adsorption process and interaction between adsorbate and sorbent surfaces [40,41,42]. Calix[4]arenes are able to bind some cations, anions and neutral molecules to form inclusion complexes in aqueous solution. Intermolecular forces that are characterized as weak, such as of ion-dipole, dipole-dipole, dipole-induced dipole, van der Waals, electrostatic interaction, hydrogen bonding, and hydrophobic interaction (CH–π or π–π) have been evidenced to cooperatively contribute to the inclusion complexation of guest molecules with calixarenes [43]. In our case, the complex stability of calixarenes was attributed to a great extent to van der Waals forces and hydrophobic interaction (CH–π) for all materials. In addition, electrostatic interaction could be considered when MCM-TDI-C4S material was used as adsorbent (the presence of sulfonated anion) (Scheme 1).

### 2.4. Effect of Initial TBT Concentration

**Figure 6:**
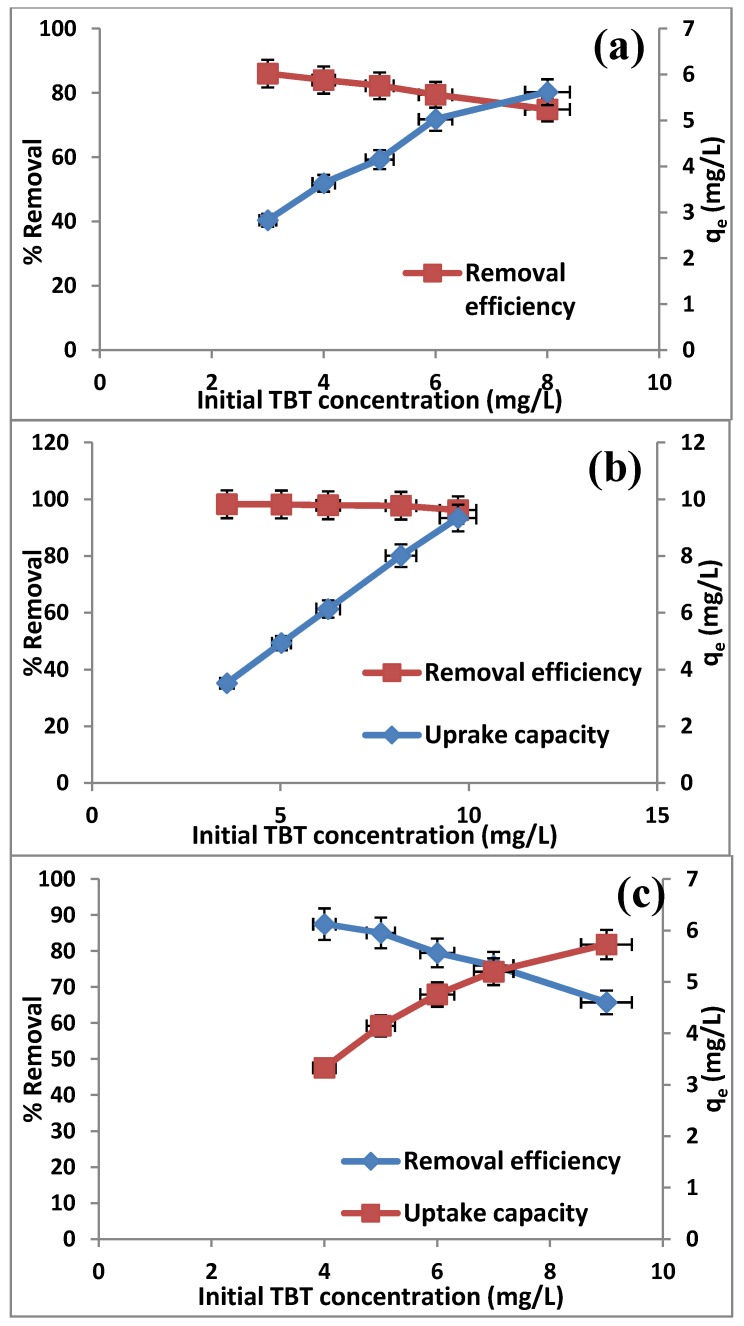
Effect of initial TBT concentration on the TBT removal efficiency and uptake capacity by MCM-TDI-C4 (**a**), MCM-TDI-PC4 (**b**) and MCM-TDI-C4S (**c**).

The adsorption experiments at initial TBT concentrations from 3 to 10 mg/L were also performed with maintaining the adsorbent amount of 0.01g at optimum pH, and the results are represented in Figure 6. The results indicate that the percentage removal [Equation (2)] decreased and the adsorption capacity [Equation (3)] increased with an increase in the initial TBT concentration for all adsorbents (at 30 °C). The decrease in the percentage removal of TBT can be explained with the fact that all adsorbents have a limited number of active sites, which would have become saturated above a certain concentration. The decrease in the percentage removal was significant in the case of MCM-TDI-C4 and MCM-TDI-C4S. This indicates that the MCM-TDI-PC4 materials have high active sites for adsorption of TBT compared to other materials.

### 2.5. Effect of Solution Temperature

**Figure 7:**
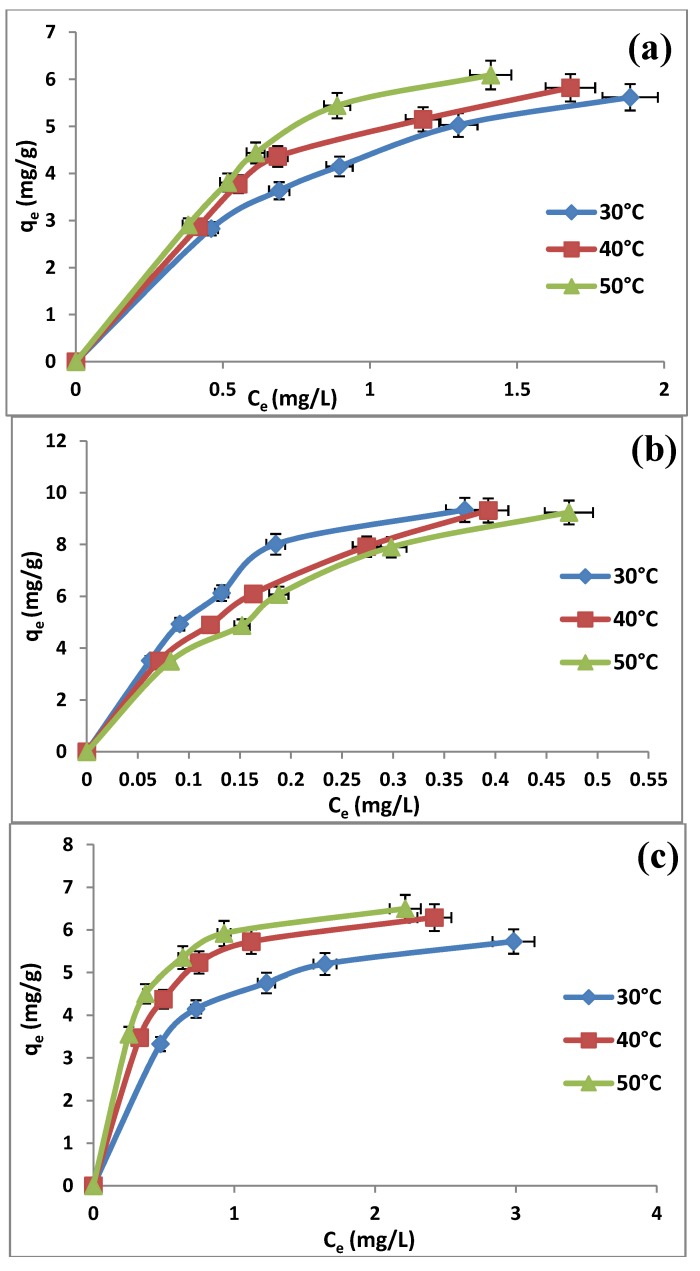
Adsorption isotherm for TBT on MCM-TDI-C4 (**a**), MCM-TDI-PC4 (**b**) and MCM-TDI-C4S (**c**) at different temperatures.

The temperature influences the adsorption equilibrium and its variations produce a displacement from or toward the phase adsorbed. Also, an increase in temperature generally improves the solubility of the molecules (if in the liquid phase) and their diffusion within the pores of the adsorbent materials [44]. The effect of varying temperature on the adsorption of TBT by MCM-TDI-C4, MCM-TDI-PC4 and MCM-TDI-C4S was examined under temperatures of 30, 40 and 50 °C, and the experimental results are illustrated in Figure 7. 

The bigger adsorptive capacities of TBT were observed in the higher temperature range for MCM-TDI-C4 and MCM-TDI-C4S materials. This was due to the increasing tendency of TBT to adsorb from the solution to the interface with an increase in temperature. The continuous increase in the adsorption capacity indicated that the adsorption process was endothermic. The TBT adsorption uptakes by MCM-TDI-PC4 were found to decrease with increase in the solution temperature from 30 to 50 °C. The decrease in adsorption capacity with the increase in temperature is known to be due to the enhancement of the desorption step in the sorption mechanism. It is also due to the weakening of sorptive forces between the active sites on the prepared material and TBT, and also between adjacent TBT molecules on the sorbed phase [45]. This result indicated that the adsorption reaction of TBT adsorbed by MCM-TDI-PC4 is an exothermic. A further discussion of temperature in light of thermodynamic parameters is provided in Section 2.7.

### 2.6. Adsorption Isotherms

In the current study, two-parameter isotherms proposed by Freundlich, Langmuir and Dubinin-Radushkevitch (D–R), and three-parameter isotherms proposed by Redlich–Peterson and Koble–Corrigan were examined with the equilibrium data obtained from the experiments. The fitted parameters for two and three-parameter models, as well as the correlation coefficient (R^2^) are summarized in Table 2.

**Table 2:** Isotherm constants and correlation coefficient of determination for various adsorption isotherms for the adsorption of TBT onto MCM-TDI-C4 (**a**), MCM-TDI-PC4 (**b**) and MCM-TDI-C4S (**c**).

Adsorbent	Adsorption isotherm	Isotherm parameter	Temperature		
			30	40	50
**(a)**	Freundlich	K_F_ (L/g)	4.2815	4.7588	5.4225
		n	2.0354	2.1322	1.7879
		R^2^	0.9845	0.9309	0.9307
	Langmuir	q_m_ (mg/g)	8.4104	9.1659	12.1212
		K_L_ (L/mg)	1.1009	1.1693	0.8629
		R^2^	0.9992	0.9609	0.9719
	Temkin	A_T_	2.0189	2.0038	2.4706
		K_T_ (L/mg)	8.8305	11.3189	9.1593
		R^2^	0.997	0.9735	0.9704
	Dubinin-Radushkevitch	q_d_ (mg/g)	6.0376	6.4967	7.3075
		β (mol^2^/kJ^2^)	3.01 × 10^−3^	2.6 × 10^−3^	2.6× 10^−3^
		E (kJ/mol)	12.8689	13.6495	13.6495
		R^2^	0.9823	0.9898	0.9957
	Redlich–Peterson	g	0.9301	0.9689	0.8918
		B_R_(L/mg)	1.2792	1.3577	1.1522
		A_R_(L/g)	10.0043	11.5734	11.9312
		R^2^	0.9986	0.9554	0.924
	Koble–Corrigan	p	1.0176	2.1208	1.8879
		A_K_	9.4697	35.4609	30.8642
		B_K_	1.1468	5.9397	4.4907
		R^2^	0.9992	0.9959	0.9986
**(b)**	Freundlich	K_F_ (L/g)	17.8525	16.4626	15.0038
		n	1.8205	1.7422	1.7382
		R^2^	0.9365	0.9928	0.9814
	Langmuir	q_m_ (mg/g)	16.4204	14.2653	13.9276
		K_L_ (L/mg)	4.5111	4.5226	3.9888
		R^2^	0.9875	0.9975	0.9852
	Temkin	A_T_	3.3637	3.4397	3.427
		K_T_ (L/mg)	48.1922	36.8125	31.3889
		R^2^	0.9731	0.9945	0.9832
	Dubinin-Radushkevitch	q_d_(mg/g)	12.8559	11.8829	11.4662
		β (mol^2^/kJ^2^)	6.7 × 10^−4^	1.01 × 10^−3^	1.01 × 10^−3^
		E (kJ/mol)	27.2991	22.2896	22.2896
		R^2^	0.9861	0.9937	0.9788
	Redlich–Peterson	g	0.998	0.9992	0.9883
		B_R_ (L/mg)	4.8264	4.4406	3.8395
		A_R_ (L/g)	76.1627	64.2147	55.2684
		R^2^	0.9657	0.9943	0.971
	Koble–Corrigan	p	1.383	0.849	0.742
		A_K_	238.0952	40.4858	25.4453
		B_K_	21.0238	2.1053	0.9033
		R^2^	0.9958	0.9988	0.9897
**(c)**	Freundlich	K_F_ (L/g)	4.3631	5.2396	5.6572
		n	3.4507	3.4602	3.7439
		R^2^	0.9526	0.8943	0.8919
	Langmuir	q_m_ (mg/g)	6.6577	7.593	7.5757
		K_L_ (L/mg)	2.1396	2.6552	3.6666
		R^2^	0.9952	0.9881	0.9875
	Temkin	A_T_	1.2949	1.3988	1.3319
		K_T_ (L/mg)	30.9003	45.1689	73.7734
		R^2^	0.9801	0.9367	0.9366
	Dubinin-Radushkevitch	q_d_ (mg/g)	5.7552	6.5463	6.7282
		β (mol^2^/kJ^2^)	2.3 × 10^−3^	1.6 × 10^−3^	1.0 × 10^−3^
		E (kJ/mol)	14.5919	17.2655	22.2896
		R^2^	0.9866	0.9993	0.9979
	Redlich–Peterson	g	0.9893	0.999	0.9937
		B_R_ (L/mg)	2.2506	2.8092	3.9357
		A_R_ (L/g)	14.7273	20.9121	29.1639
		R^2^	0.9989	0.9968	0.9972
	Koble–Corrigan	p	1.1583	1.4241	1.4278
		A_K_	16.8919	35.5872	54.6448
		B_K_	2.6892	5.3594	8.1366
		R^2^	0.9964	0.9993	0.9984

**Figure 8:**
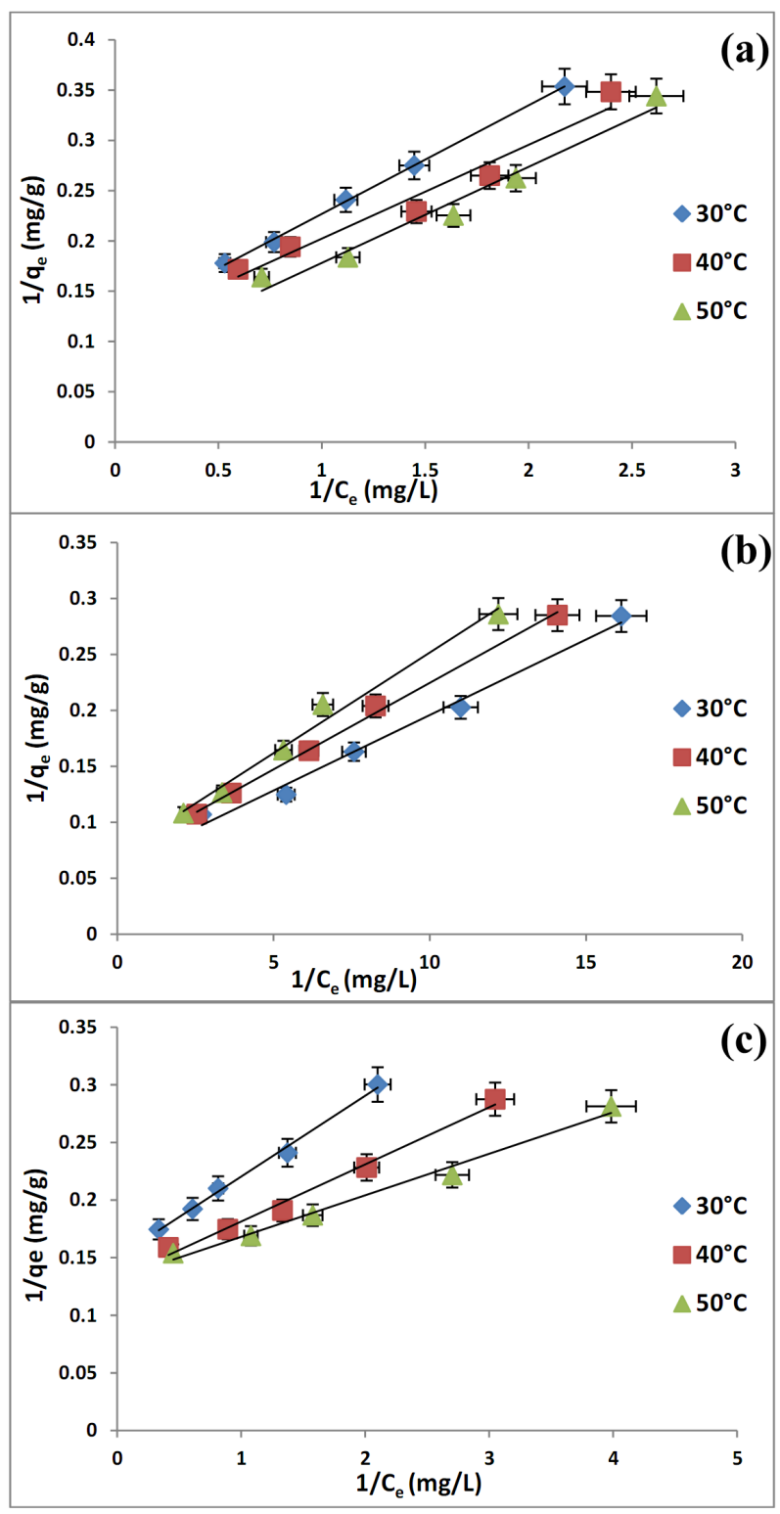
Langmuir isotherm of TBT adsorbed onto MCM-TDI-C4 (**a**), MCM-TDI-PC4 (**b**) and MCM-TDI-C4S (**c**).

**Figure 9:**
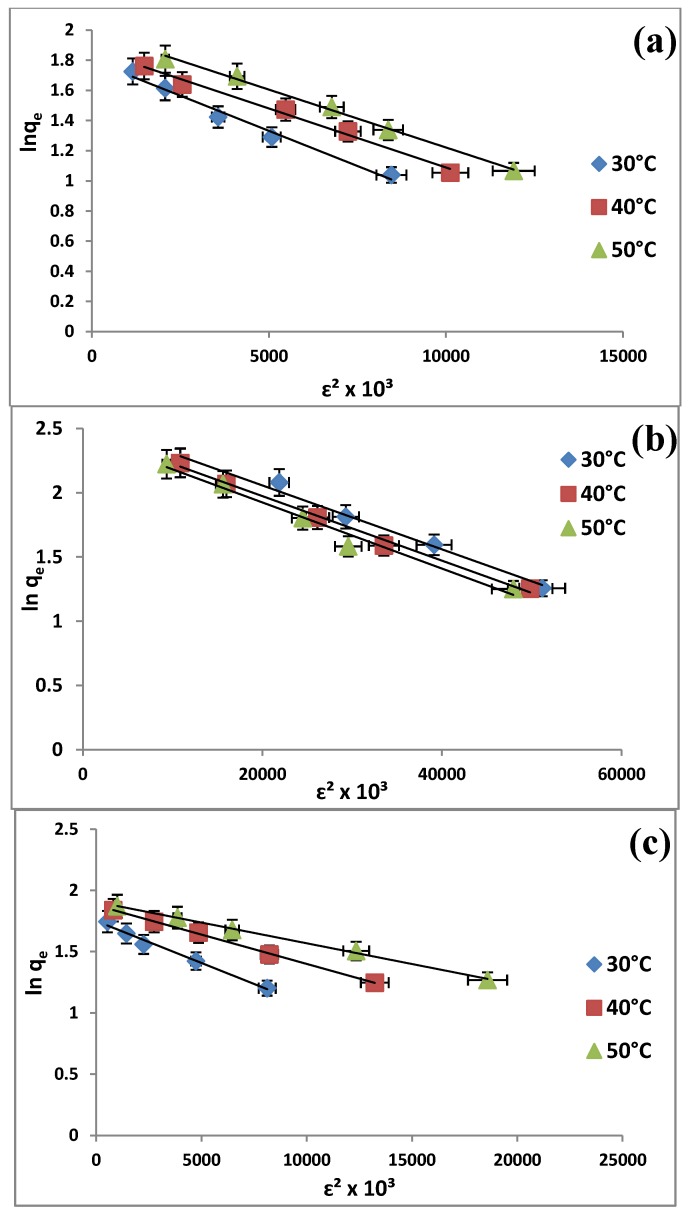
Dubinin-Radushkevitch isotherm of TBT adsorbed onto MCM-TDI-C4 (**a**), MCM-TDI-PC4 (**b**) and MCM-TDI-C4S (**c**).

It is found that two-parameter models, both of Langmuir (Figure 8) and Dubinin-Radushkevitch (Figure 9) equations are suitable for correlating the isotherm data due to a high R^2^ (>0.98) (Table 3) and the Langmuir equation is more suitable for representing the equilibrium adsorption. The monolayer adsorption capacities of the adsorbents from Langmuir isotherm model are found to be of the order: MCM-TDI-PC4 > MCM-TDI-C4> MCM-TDI-C4S. The difference of q_m_ derived from the Langmuir and q_d_ from D–R models may be attributed to the different definition of maximum adsorption capacity in two models. In Langmuir model, q_m_ represents the maximum adsorption of TBT at monolayer coverage, whereas q_d_ represents the maximum adsorption of TBT at the total specific micropore volume of the adsorbent in D–R model. The values of n from Freundlich isotherm represent a favourable adsorption condition. In addition, a higher values of n in case of MCM-TDI C4S indicates the formation of relatively strong bond between the TBT and adsorbent [46].

**Table 3:** Thermodynamic parameters of TBT adsorption.

	T (°C)	Thermodynamic parameters		
		ΔG° (kJ/mol)	ΔH° (kJ/mol)	ΔS° (J/mol K)
	30	−4.5779	8.669	43.7192
MCM-TDI-C4	40	−5.0151		
	50	−5.4523		
	30	−10.0418	−21.3952	−37.4704
MCM-TDI-PC4	40	−9.6671		
	50	−9.2924		
	30	−36.5595	30.8066	120.7609
MCM-TDI-C4S	40	−37.7671		
	50	−38.9747		

The three-parameter isotherm model shows a better correlation with the experimental adsorption data than the two-parameter isotherm model. The calix[4]arene derivatives functional groups created on the mesoporous silica surface make the mesoporous silica surface heterogeneous and the heterogeneity depends on the different calix[4]arene derivatives. Furthermore, the porosity factors β from the Dubinin-Radushkevitch isotherm for TBT were <1 for the three adsorbents. This demonstrates the existence of micropores in addition to mesoporous that confirms the heterogeneity of the surface which arises from the pore structure as well as adsorbate adsorbent interaction. The exponent values of g from Redlich–Peterson isotherm model were = 0.9301, 0.998 and 0.9893 for MCM-TDI-C4, MCM-TDI-PC4 and MCM-TDI-C4S, respectively, signifying the fit of Langmuir model for explaining the obtained equilibrium data which inferred that the primary mechanism of the TBT adsorption process is the homogeneous uptake. The best fitting of Koble–Corrigan isotherm with the experimental equilibrium data confirmed the combination of heterogeneous and homogenous uptake for TBT through the synthesized adsorbents. Comparing the coefficients of determination of the Freundlich and Langmuir models, we infer that homogeneous uptake was the main mechanism of the TBT adsorption process. The values of p ≈ 1 for MCM-TDI-C4 and MCM-TDI-C4S revealed that the adsorption reaction stoichiometry would be 1 solute molecule per free adsorbent site [47]. These results for MCM-TDI-C4 and MCM-TDI-C4S suggested that TBT was adsorbed by a 1:1 mechanism (monolayer sorption). These results are in agreement with the previous results from Langmuir and Redlich–Peterson isotherm models.

**Figure 10:**
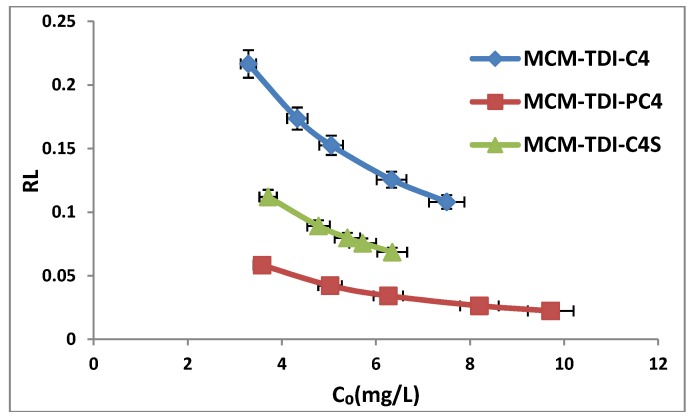
Values of R_L_ for adsorption of TBT onto MCM-TDI-C4, MCM-TDI-C4S and MCM-TDI-PC4.

Furthermore, it was observed that the separation factor R_L_ at 30 °C (Figure 10) are between zero and one. which indicated these three adsorption processes are favourable over the range of initial TBT concentrations that were applied, the degree of favourability is in the order of MCM-TDI-PC4 > MCM-TDI-C4S > MCM-TDI-C4. The *R*_L_ values decrease with increase in initial TBT concentration, indicating that the adsorption was more favorable at higher TBT concentration.

The mean free energies of adsorption E calculated from Dubinin-Radushkevitch isotherm are less than 16 kJ/mol, indicating that the TBT adsorption on MCM-TDI-C4 took place through physical adsorption at 30 °C and higher than 16 kJ/mol, which implies that the type of adsorption appears to be chemical on MCM-TDI-PC4 and also could be in case of MCM-TDI-C4S. Two adsorption mechanisms, *i.e.*, van der Waals forces and hydrophobic interactions, involve in the adsorption of the TBT. Because of calix[4]arenes containing *tert*-butyl groups which provide hydrophobic cavities capable of hydrophobic interactions with *n*-butyl chains.

### 2.7. Adsorption Thermodynamic

**Figure 11:**
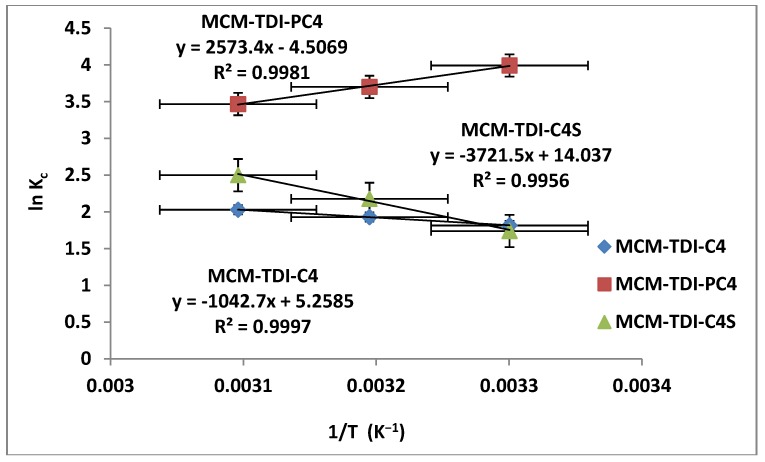
Plot of lnK_c_ *versus* 1/T for MCM-TDI-C4, MCM-TDI-C4S and MCM-TDI-PC4.

The calculated values of ΔH°, ΔS° and ΔG° for the adsorption of TBT on MCM-TDI-C4, MCM-TDI-PC4 and MCM-TDI-C4S are shown in Table 3 (Figure 11). An important difference between the three adsorbents is the magnitude values of thermodynamic parameters. The higher magnitude values of ΔH°, ΔS° and ΔG° for MCM-TDI-C4S compared to MCM-TDI-C4 and MCM-TDI-PC4 may be related to the difference in the mechanisms of adsorption. The TBT adsorption by MCM-TDI-C4S occurred through strong electrostatic interactions, thus the process can lead to significant modifications of the surface groups and of the TBT molecule. In contrast, the TBT adsorption by MCM-TDI-PC4 and MCM-TDI-C4 was more related to the hydrophobic interactions and the process was just the formation of a dense layer of molecules on the surface of the adsorbents material.

The positive ΔH° values for MCM-TDI-C4 and MCM-TDI-C4S and the negative ΔH° value for MCM-TDI-PC4 indicated that the adsorption process was endothermic and exothermic in nature, respectively, which is consistent with the results obtained earlier in the effect of temperature in Section 2.5. In addition, the magnitude values of ΔH° indicate that the adsorption of TBT on MCM-TDI-C4 was a physisorption, and the adsorption of TBT on MCM-TDI-PC4 and MCM-TDI-C4S was chemisorption. Additionally, the energy value taken from the D-R model was consistent with these results.

The ΔS° positive values for MCM-TDI-C4 and MCM-TDI-C4S indicate the increased randomness at the interface of solid-solution during the process of sorption. Meanwhile, the negative ΔS° value suggests a decrease in the randomness at the solid/solution interface during the adsorption of TBT onto MCM-TDI-PC4. Furthermore, the solubility of MCM-TDI-C4S adsorbent plays an important role in the magnitude values of ΔS°. As in an aqueous solution, water molecules easily wet hydrophilic MCM-TDI-C4S adsorbent, TBT must received some heat and replace the water molecules and then it could be adsorbed on the MCM-TDI-C4S adsorbent surface. The action is called “solvent replacement” [48]. For TBT molecule with larger molar volume than water molecule, the number of water molecule replaced was larger than that of TBT molecule adsorbed. Hence, the solvent replacement results in the increase of entropy.

The ΔG° values for all adsorbents were negative, which reflectd the spontaneous nature of the adsorption processes. The spontaneous adsorption of TBT onto MCM-TDI-PC4 was mainly driven by the enthalpy change, while MCM-TDI-C4 and MCM-TDI-C4S was mainly driven by the entropy change. Compared to MCM-TDI-C4 and MCM-TDI-PC4, the largest absolute value of ΔG° for MCM-TDI-C4S suggests the most spontaneous nature of the adsorption processes of TBT to MCM-TDI-C4S. It was probably attributed to the electrostatic attraction that occurs at the upper rim of the calix[4]arene (the presence of sulfonated anion) in addition to the hydrophobic interaction with the host cavity (CH–π). The values of ΔG° for MCM-TDI-C4 and MCM-TDI-C4S were found to increase as the temperature increased, indicating high driving force and hence resulting in high adsorption capacity.

## 3. Experimental

### 3.1. Materials and Instrumentation

The chemicals used are as follows: mesoporous silica (Aldrich, MO, USA, surface area 993 m^2^/g, average diameter of 2.9 nm) as silica source. Calix[4]arene (C_28_H_24_O_4_, Acros, Morris Plains, NJ, USA) and *p-tert*-butylcalix[4]arene (C_44_H_56_O_4_, Fluka, St. Louis, MO, USA) were the organic modifiers. Toluene 2,4-diisocyanate (TDI, C_9_H_6_N_2_O_2_, Aldrich, Buches SG, Switzerland) was the organic linker. Triethylamine (C_6_H_15_N, SAFC, Steinheim, Germany) was used as catalyst. Di-*n*-butylamine (C_8_H_19_N, Acros) and hydrochloric acid (HCl, Fisher, Loughborough, UK) were used for the determination of isocyanate groups. Dichloromethane (CH_2_Cl_2_, Sigma Aldrich, Steinheim, Germany), chlorosulfonic acid (HSO_3_Cl, Merck, Hohenbrunn, Germany) and methanol were utilized for the synthesis of p-sulfonatocalix[4]arene as described in the literature [49]. Toluene (Fisher, dried before use by using molecular sieves), ethanol (Fisher) and acetone (Fisher) were used as solvents. Water was purified using Milli-Q purification equipment. For sorption experiments tributyltin chloride (C_12_H_27_ClSn Aldrich), concentrations were adjusted by successive dilutions with Milli-Q water of an 8.42 mM solutions in methanol stored at 4 °C in the dark. A methanolic stock solution of organotins compound was used because of the very low organotins solubility in water.

Fourier transform infrared spectra (FTIR) were recorded on a Perkin Elmer FTIR Specrum RX1 ATR (Waltham, MT, USA) with a KBr pellet technique. Nitrogen adsorption–desorption experiments were carried out at 77.40 K on a Quantachrome Autosorb Automated Gas Sorption system (Boynton Beach, FL, USA). The Brunauer–Emmett–Teller (BET) surface area (S_BET_) was calculated from the linearity of the BET equation while the volume and pore diameters were calculated from the pore size distribution curves using the Density Functional Theory DFT method.

An Agilent Technology 7500 series ICP-MS (Santa Clara, CA, USA) was used for the determination of DBT in aqueous solutions. A series of Sn standard solutions were used to construct the calibration curve, for which a good linear relationship was observed. 

### 3.2. Preparation of Functionalized Mesoporous Silica

Mesoporous silica functionalized with calix[4]arene derivatives were prepared according to our previous work [50]. Briefly, mesoporous silica MCM-41 (5.0 g, 150 °C, 4 h) and excess of TDI (200 mL, 1.41 mol, dried with molecular sieves for 24 h) were mixed using a magnetic stirrer and the functionalization was performed in a dry nitrogen atmosphere at 80 °C for 4 h. In order to remove all the substances physically adsorbed on the surface of the particles, the sample of mesoporous silica -TDI was separated by centrifugation and sequentially rinsed with toluene and acetone. The sample was marked as MCM-TDI. 2.0 mmol of calix[4]arene derivatives (with calix[4]arene, *p-tert*-butyl-calix[4]arene and *p*-sulfonatocalix[4]arene), was added into the MCM-TDI (1 gm) suspension with toluene (dried by molecular sieves for 24 h). Subsequently, triethylamine was added and the reaction temperature was kept at 80 °C for 24 h under stirring. Then the particles were separated by centrifugation, washed with toluene and acetone and dried overnight. The sample was marked as MCM-TDI-C4, MCM-TDI-PC4 and MCM-TDI-C4S for calix[4]arene, *p-tert*-butylcalix[4]arene and *p*-sulfonatocalix[4]arene, respectively. 

### 3.3. Adsorption Isotherms

The adsorption experiments were conducted by the inclusion of a specific amount of adsorbent (0.01 g) into 50 mL-Teflon reactors (FEP, Nalgene) containing diluted solutions (10 mL) of known concentrations. The Teflon reactors were sealed and inserted in a water bath shaker (Wise Bath WSB-18, Wertheim, Germany) and agitated at 180 rpm for 120 min at 30, 40 and 50 °C, where the solution was adjusted to optimum pH. Then, Teflon reactors were taken out of the shaker, filtered through 0.45 µm membrane filter and the TBT final concentration in the solution was calculated through ICP-MS. The batch reactors were initially cleaned with Decon 90 and 10% nitric acid, and later washed with Milli-Q water. TBT removal efficiency in aqueous solution was calculated by the equation:

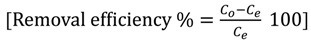
(2)

TBT uptake, q_e_ (mg/g) by the adsorbents was determined, as follows:

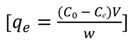
(3)
where C_o_ and C_e_ are the initial and final concentration of the TBT solution (mg/L), respectively, V is the volume of the solution (L), and w is the mass of sorbents (g).

The impact on TBT adsorption by the initial pH on adsorbents was conducted with 0.01 g adsorbents at the temperature of 30 °C. The range of pH for the solution was adjusted between 3 and 8.

### 3.4. Mathematical Modelling

#### 3.4.1. Isotherm Models

The adsorption isotherm is very useful to describe how the adsorption molecules distribute between the liquid phase and the solid phase when the adsorption process reaches an equilibrium state. The adsorption isotherm data were fitted by two parameter isotherms (Freundlich, Langmuir and Dubinin-Radushkevitch) and three parameters isotherms (Redlich–Peterson and Koble–Corrigan). The parameters obtained from the models provide important information on the sorption mechanism and the surface property and affinity of the adsorbent.

The Freundlich isotherm is applicable to both monolayer (chemisorption) and multilayer adsorption (physisorption) [51]. The linear form of Freundlich equation is expressed as:

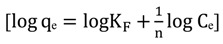
(4)
where q_e_ (mg/g) is the amount of solute adsorbed per unit weight of adsorbent at equilibrium, C_e_ (mg/L) is the equilibrium liquid phase concentration, and K_F_ (L/g) is roughly an indicator of the adsorption capacity and the slope 1/n, ranging between 0 and 1, is a measure for the adsorption intensity or surface heterogeneity [52].

The Langmuir isotherm assumes monolayer adsorption on a uniform surface with a finite number of adsorption sites [53]. The linear form of the Langmuir isotherm model is described as:

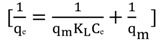
(5)
where K_L_ (L/mg) is the Langmuir constant related to the energy of adsorption and q_m_ is the maximum adsorption capacity (mg/g).

The essential characteristics of the Langmuir isotherm can be expressed in terms of a dimensionless separation factor (R_L_) [54] which is defined by:

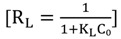
(6)
where K_L_ is the Langmuir constant and C_0_ is the initial concentration (mg/L) of adsorbate. The value of R_L_ indicates the type of the isotherm to be either unfavorable (R_L_ > 1), linear (R_L_ = 1), favorable (0 < R_L_ <1) or irreversible (R_L_ = 0).

Dubinin-Radushkevitch (D-R) isotherm model is a semi-empirical equation where the adsorption is characterized by multi-layer involving van der Waals forces and is suitable for physical process of adsorption [55]. The expression of linear form of D-R isotherm model is represented as:

[lnq_e_ = lnq_d_ - βε^2^] (7)

where q_d_ is the D-R constant which refers to maximum adsorption capacity (mg/ g), β (mol^2^/kJ^2^) is the constant related to free energy and ε is the Polanyi potential which is defined as:

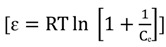
(8)
where R is the gas constant (8.31 J/mol K) and T is the absolute temperature. The constant β gives the mean free energy E by using the relationship:

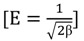
(9)
E values that are between 1–16 kJ/mol facilitate physical adsorption while values over 16 kJ/mol facilitate chemisorptions [56,57].

The abilities of the three-parameter equations, Redlich– Peterson and Koble–Corrigan isotherms, to model the equilibrium adsorption data were also examined. Redlich- Peterson brought forward an empirical equation which combines both Langmuir and Freundlich equations elements and the adsorption mechanism is described as a hybrid which is distinct from an ideal monolayer adsorption. The linear form of Redlich–Peterson isotherm model is expressed as:

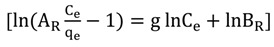
(10)
where q_e_ is the concentration of solid phase sorbate in equilibrium (mg/g), C_e_ is the concentration of liquid phase sorbate in equilibrium (mg/L), A_R_ (L/g) and B_R_ (L/mg) are R-P isotherm constants, and g is the exponent which lies between 0 and 1. The three isotherm constants are evaluated through the use of a Solver add-in function of the Microsoft Excel [58].

Koble-Corrigan model is described as a three-parameter empirical model that represents the equilibrium adsorption data [59]. Similar to the above isotherm, it is a combination of the Langmuir and Freundlich isotherm models and the linear form of this module is represented by [Equation (11)].

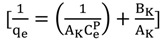
(11)
where, A_K_, B_K_ and p are the Koble-Corrigan parameters, respectively. The Koble-Corrigan model’s three isotherm constants are also evaluated through the use of a Solver add-in function of the Microsoft Excel.

#### 3.4.2. Adsorption Thermodynamic

The thermodynamic parameters can be determined from the thermodynamic equilibrium constant K_c_. The standard Gibbs free energy ΔG° (kJ/ mol), standard enthalpy change ΔH° (kJ/ mol), and standard entropy change ΔS° (J/mol K) were calculated using the following equations:

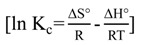
(12)

[∆G° = ∆H° - T∆S°]
(13)

where R (8.314 J/ mol K) is the gas constant, T (K) is the absolute temperature and *K*_c_ (L/g) is the standard thermodynamic equilibrium constant defined by q_e_/C_e_.

## 4. Conclusions

The adsorption of TBT on MCM-TDI-C4, MCM-TDI-PC4 and MCM-TDI-C4S from aqueous solutions is efficient, and the maximum adsorption capacities were measured to be 12.1212, 16.4204 and 7.5757 mg/g, respectively. The difference in the upper rim of the calix[4]arene at the surface of MCM-TDI-C4, MCM-TDI-PC4 and MCM-TDI-C4S reflects directly on adsorption capacities of these materials. The high adsorption capacity of MCM-TDI-PC4 is attributed to hydrophobic interactions occurring between the *n*-butyl chain of the butyl group in TBT and the tributyl groups on the calix[4]arene upper rim, and to the broad pore size distribution. The two parameter and three parameter models were used to describe the isotherms and the Koble–Corrigan model was shown to provide the best fitting. This indicates that a combination of heterogeneous and homogeneous uptake occurred. The calculated values of separation factors and Gibbs free energy changes indicated that the adsorption processes were favourable, feasible and spontaneous. Furthermore, from thermodynamic studies MCM-TDI-C4S was the most favourable adsorbent compared to MCM-TDI-C4, MCM-TDI-PC4, which can probably be attributed to the hydrophobic interaction with the host cavity (CH–π) and the electrostatic attraction that occurs at the upper rim of the calix[4]arene (sulfonate groups). The spontaneous adsorption of TBT onto MCM-TDI-PC4 was driven mainly by enthalpy change while driven mainly by entropy change onto MCM-TDI-C4 and MCM-TDI-C4S.
